# Development of Lasing Silica Microsphere for High-Speed DNA Molecular Detection

**DOI:** 10.3390/s24186088

**Published:** 2024-09-20

**Authors:** Chan Seok Jun, Wonsuk Lee

**Affiliations:** 1 Department of Material Science and Engineering, Korea University, Seoul 02841, Republic of Korea; 2 Nanophotonics Research Center, Korea Institute of Science and Technology, Seoul 02792, Republic of Korea

**Keywords:** DNA, optofluidic laser, silica microsphere, molecular detection, fluorescence

## Abstract

Laser and molecular detection techniques that have been used to overcome the limitations of fluorescent DNA labeling have presented new challenges. To address some of these challenges, we developed a DNA laser that uses a solid-state silica microsphere as a ring resonator and a site for DNA-binding reactions, as well as a platform to detect and sequence target DNA molecules. We detected target DNA using laser emission from a DNA-labeling dye and a developed solid-state silica microsphere ring resonator. The microsphere was sensitive; a single base mismatch in the DNA resulted in the absence of an optical signal. As each individual microsphere can be utilized as a parallel DNA analysis chamber, this optical digital detection scheme allows for high-throughput and rapid analysis. More importantly, the solid-state DNA laser is free from deformation, which guarantees stable lasing characteristics, and can be manipulated freely outside the solution. Thus, this promising advanced DNA laser scheme can be implemented on platforms other than optofluidic chips.

## 1. Introduction

The completion of the human genome map provides genomic information that can be used to gain clinically useful insights into an individual’s genetic susceptibility to various diseases, such as certain cancers [1,2,3,4,5,6,7,8,9]. In addition, the analysis and detection of DNA sequences play a vital role in many areas of life sciences and medicine, including disease diagnosis and rapid epidemiological surveys during infectious disease outbreaks, such as the recent COVID-19 pandemic. To date, DNA sequence analysis has relied on fluorescent DNA labeling, as achieved through real-time polymerase chain reaction (PCR) or DNA arrays [10,11,12,13,14,15,16].

However, traditional fluorescence-based techniques are associated with complicated optical measurements and time-consuming processes. To overcome these limitations, laser and molecular detection techniques have been applied to DNA analyses [17,18,19,20,21,22,23,24]. These laser-based techniques increase speed and throughput as well as simplify the analysis process. By exploiting the nonlinear optical phenomenon with the laser threshold, the analog fluorescence signal analysis was replaced with digital analysis, which allows for DNA analysis in the presence or absence of laser oscillations, greatly reducing analysis time and simplifying optical signal analysis.

Optofluidic technology, which utilizes liquid gain media, is an optimal platform for implementing laser-based DNA analysis/detection technologies [19,22,25,26]. Early DNA lasers were implemented using glass capillaries as fluidic channels and their cross sections as ring resonators [18,19,20,25]. Ring resonators have a naturally high Q-factor, which makes it easier to generate laser oscillations from DNA and fluorescent dyes. Their intrinsic multimode laser output is not disadvantageous in DNA analysis, since the detection is carried out only with or without laser oscillations. Thus, the use of the optofluidic ring resonator (OFRR) platform would optimize DNA detection. However, to minimize the analysis time, which is the main advantage of laser-based DNA detection, a platform capable of parallel and simultaneous multiple-sample analysis is required, which is difficult to implement in a glass capillary configuration. Therefore, there have been proposals for creating DNA and dye microdroplet samples, with each drop serving as a sample reaction chamber and, simultaneously, as a spherical ring resonator [26,27]. In such a platform, each microdroplet can be analyzed in parallel as an independent DNA sample, enabling high-speed and high-throughput DNA analysis. However, this presents limitations: a microdroplet can only be developed in a fluid channel containing two immiscible fluids, and its shape is not fixed outside of the controlled experimental conditions; thus, the laser’s characteristics are not reliable. The concentration of DNA/dye molecules also must be kept relatively high inside the microdroplet to obtain laser oscillation with descent optical pump energy.

To address these challenges, in this study, we developed a DNA laser that uses a solid-state silica microsphere as a ring resonator and a site for DNA-binding reactions. In addition, we developed a platform to detect and sequence target DNA molecules. The single-stranded DNA probe functionalized on the surface of the silica microspheres effectively separated and detected the target DNA from the control samples. The interaction of the fluorescent dye attached to the DNA and the evanescent field of the silica resonator successfully triggered the laser, enabling a novel method for digital ultrafast DNA sequencing.

## 2. Materials and Methods

**Figure 1:**
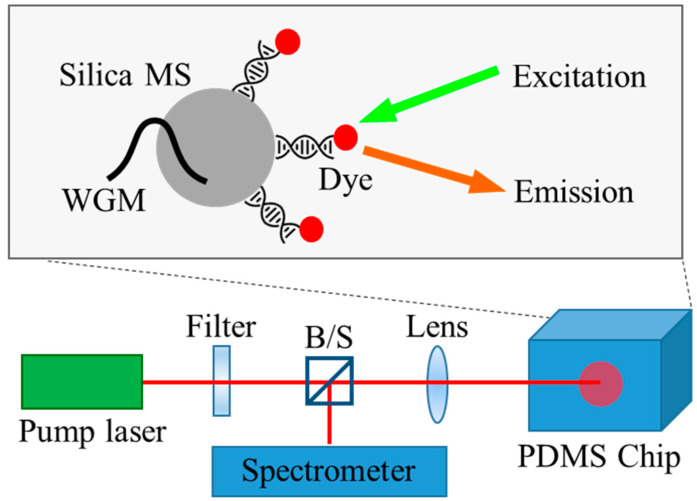
Silica microspheres support the internal WGM and the interaction of the outer evanescent field with the DNA/dye attached to their surface, providing feedback for laser oscillation. The microspheres are injected into a fluidic channel fabricated on a PDMS chip. The optical pump and laser oscillation measurements are performed simultaneously through a confocal setup.

The microspheres were fabricated by the silanization of acid-washed silica beads (100 μM in diameter; Benchmark Scientific, Sayreville, NJ, USA). The silica beads were soaked in (3-aminopropyl)-trimethoxysilane (3-APTMS, 5% in methanol) for 30 min. The microspheres were rinsed with ethanol, cured at 110 °C for 1 h, and cooled to room temperature. To immobilize the DNA probe molecules, the microsphere surface was functionalized with bis(sulfosuccinimidyl)-suberate (BS3), a homofunctional amine-to-amine crosslinker. BS3 was dissolved in phosphate-buffered saline (PBS) to a concentration of 0.1 mg/mL, and the silica beads were immersed in the solution for 30 min. See Figure 1.

Next, the beads were rinsed with PBS, and the functionalized silica beads were incubated with streptavidin; the silica beads were soaked in 1 mg/mL streptavidin/PBS solution for 30 min. The streptavidin concentration was high to ensure that the silica surface was sufficiently covered with the molecules. The streptavidin-incubated silica beads were rinsed with PBS, DI water, and Tris-acetate-EDTA (TAE)/12.5 mM MgCl_2_ buffer solution.

The single-stranded DNA (ssDNA) molecules that acted as the probe DNA were 21 bases long, and their sequence was randomized and biotinylated at the 5′ end. The ssDNA molecules used in the experiments were purchased from Integrated DNA Technologies, and their sequences are listed in Table 1. Probe DNA was dissolved in TAE/MgCl_2_ buffer solution at a concentration of 1 μM, and the silica beads were immersed in the solution for 30 min. This process allows the probe DNA molecules to attach to the fiber surface via the well-known streptavidin–biotin bond, resulting in a microsphere capable of detecting the target DNA molecule that complementarily binds to the probe DNA molecules.

The DNA probe microspheres were placed in a DNA-containing solution with a sequence complementary to the probe ssDNA, labeled with Cy3 dye at the 3′ end, and subjected to a hybridization process. The concentration of Cy3 dye-labeled DNA molecules to be detected was 1 μM, the same as that of the probe DNA, and the microspheres were soaked in the solution for 20 min to ensure hybridization of the DNA. This concentration is more than two orders of magnitude lower than that used in previous studies [27], wherein microdroplets in solution were used to generate laser oscillations. This is important as it proves that this method is feasible even when the amount of DNA sample to be detected/analyzed is significantly small. The Cy3 dye-labeled DNA microspheres were then separated by rinsing and drying, dissolved in TAE/MgCl_2_ buffer solution, and injected into a fluidic channel (2 mm wide and 1 mm high) made of PDMS on a glass substrate.

The PDMS optofluidic chip was then excited from the bottom glass substrate using a pump laser light source (continuum, optical parametric oscillator, 518 nm wavelength, 5 ns pulse width, and 20 Hz repetition rate) through a confocal setup. In the 520–630 nm wavelength band, where pump and laser oscillations occur, the refractive index of silica is approximately 1.46, which is higher than that of the surrounding medium (~1.33), which is mainly composed of water. This ensures that the light is confined to the whispering gallery mode (WGM) inside the DNA microsphere. The WGM has an evanescent field outside of the microsphere, which receives feedback from the interaction of this field with the externally immobilized Cy3 dye, enabling laser oscillation. Adapting calculations from previous study [28,29], the energy residing outside surface of the microsphere is approximately 12% of the total energy, assuming a perfectly shaped, flawless glass microsphere. Although the silica beads used in this experiment are not flawless as in the calculation, we can still expect enough light–gain medium interaction and a decent Q-factor for the laser oscillations. Because the dye is a monomolecular layer coated on the outer surface of the microspheres, the fluorescence signal, unlike the laser signal, is unobservable, and the DNA detection configuration is optically truly digital. The generated laser signal was similarly collected and analyzed using a monochromator through the confocal setup.

## 3. Results

**Figure 2:**
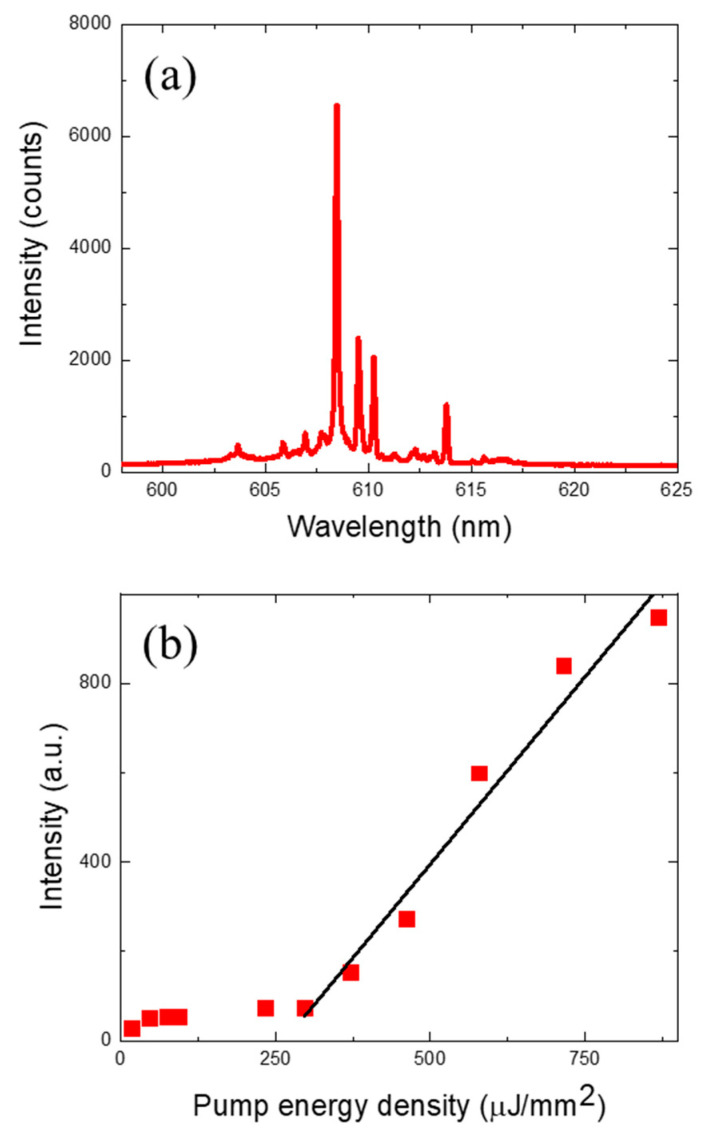
(**a**) Laser emission spectra from Cy3 dye-labeled target DNA when optically pumped with an energy density of 680 µJ/mm^2^. (**b**) Spectrally integrated laser emission intensity as a function of energy density. The laser threshold was approximately 252 µJ/mm^2^.

Representative laser emission spectra and the laser characteristics of the DNA microsphere lasers are shown in Figure 2. A PDMS fluid channel section that was approximately 1 mm long was optically pumped, and a few microspheres contained within it simultaneously generated laser oscillations. When optically pumped with an energy density of 680 µJ/mm^2^, the optical signal shown in Figure 2a was obtained, which exhibits typical multimode laser characteristics of a WGM laser with a ring resonator cavity. Unlike the two-dimensional ring resonator, the spherical ring resonator is interspersed with various TE, TM, and TEM modes. Since the laser signals from multiple microspheres were simultaneously observed with a monochromator, laser modes with random wavelengths were observed, and the free spectral range could not be derived from this spectrum. However, a clear ring resonator laser oscillation was identified, which can be seen from the optical spectrum as well as the curve in Figure 2b. The DNA laser showed a clear nonlinear laser output characteristic with a threshold of approximately 252 µJ/mm^2^. As a control experiment, we repeated the same experiment with a DNA molecule with one base mismatch in the middle of the 21 sequences as shown in Table 1 and obtained virtually no optical signal (laser) when optically pumped with the same 680 µJ/mm^2^ energy density. This proved that our DNA laser microspheres can detect the target molecule with the sensitivity of a single base mismatch in DNA of a 21-sequence length and can be analyzed in an optically digitized manner.

While previous experiments using Cy3 dye have been successful in rapidly detecting target DNA sequences with only a single laser burst, there is room for further improvement in the complexity of the entire analysis process, as the DNA sample to be analyzed must be labeled with the dye in advance, as is the case with traditional fluorescence analysis methods. In this experiment, silica microspheres with probe DNA were used in the same way, but unlabeled DNA samples were hybridized. An intercalating dye (SYTO^®^-13 Green Fluorescent Nucleic Acid Stain, Life Technologies, Carlsbad, CA, USA) was then added to the solution to stain the hybridized DNA molecules and analyzed with the same optical setup.

**Figure 3:**
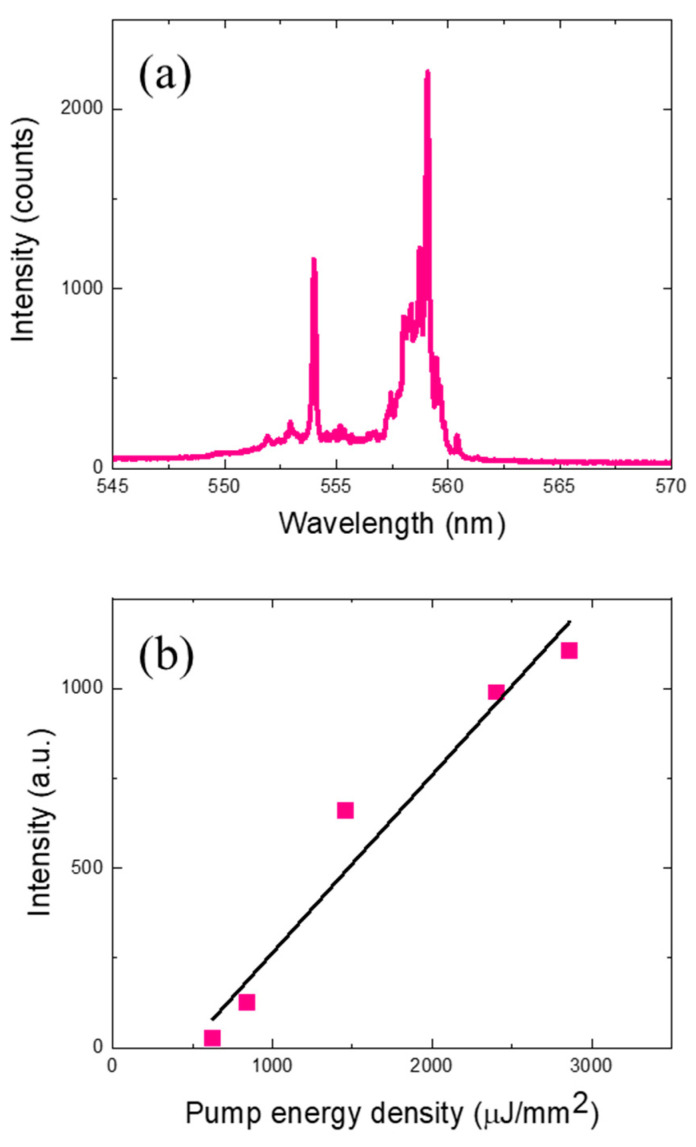
(**a**) Laser emission spectra from DNA microspheres stained with intercalating dyes without labeling. It was optically pumped with an energy density of 2.4 mJ/mm^2^. (**b**) Laser emission intensity at varying pump energy density. The laser threshold was approximately 500 µJ/mm^2^.

Figure 3a depicts the laser emission spectrum when the DNA laser microspheres with the intercalating dye were optically pumped at an energy density of approximately 2.4 mJ/mm^2^, and Figure 3b presents the spectrally integrated laser intensity as a function of pump energy density. The laser emission characteristics show the multimode spectral characteristics of a typical spherical ring resonator laser, and as with the Cy3-labeled dye, the laser threshold was measured to be approximately 490 µJ/mm^2^. Note that the pump laser wavelength has been set to 480 nm, which fits the intercalating dye absorption, and the DNA laser emission wavelength has moved accordingly. The increased laser threshold and lower laser efficiency compared to the Cy3 dye experiment is attributed to the intrinsically lower quantum efficiency of the intercalating dye and the relatively small number of dye molecules covering the microsphere surface compared to the label dye, which is attached to every hybridized DNA molecule. However, we were still unable to measure any optical signals (laser/fluorescence) from single base-mismatched DNA at the same pump energy density (2.4 mJ/mm^2^). Even though the fluorescence signal below the lasing threshold is not comparable to the laser signal, it can still be observable with more sensitive measurement settings, and this is one of proofs that the DNA and dye molecules are hybridized to the microsphere surface [Figure S1]. These observations confirm that it is possible to detect target DNA molecules at high speeds using DNA laser microspheres without any labeling process.

One of the biggest advantages of this research using solid-state silica microspheres, compared to glass capillary ring resonators or microdroplet lasers using two immiscible fluids, is the ability to manipulate the sample without fear of destroying the ring resonator shape. To demonstrate this, instead of placing the microspheres directly into the buffer solution after the target DNA molecule hybridization and dye staining, the microspheres were left to dry in ambient conditions for 24 h before repeating the same laser oscillation experiment. As expected, the lasing characteristics were virtually identical, demonstrating that the lasing DNA microspheres can be utilized on a variety of platforms that do not all go through the solution process, for ultrafast, high-throughput detection and analysis of DNA target molecules.

## 4. Discussion

By functionalizing DNA probe molecules on solid-state silica microspheres, we have proposed a method to hybridize target DNA sequences and digitally detect them via laser oscillation. Each of these DNA analyzing microspheres can be utilized as an independent DNA detection assay, enabling parallel analysis, and the detection of target DNA can be performed with or without a single laser emission, which is a quantum leap in speed and throughput compared to traditional fluorescence analysis. Unlike microdroplets, which are formed by mixing two immiscible fluids, silica microspheres in the solid state do not cause changes in laser properties through deformation. Additionally, sample consumption by our silica microsphere is significantly low, compared to the lasing microdroplets. Most importantly, the solid-state microspheres can be manipulated and controlled at will without being limited to the solution process; hence, they can be implanted not only in optofluidic chips but also in various types of biomolecular analysis/detection platforms, such as DNA microarrays. The DNA lasing microspheres developed in this study are expected to solve the problems of traditional DNA fluorescence analysis, which has limitations in terms of analysis time and throughput, and accelerate the development of life sciences and medicine through DNA sequencing.

## Figures and Tables

**Table 1:** DNA sequences of target and base-mismatch analytes.

ssDNA	Target	Base Mismatch
Sequence 1	5′-ACA ACA AAG AAC ATA CAT AGG-3′	5′-ACA ACA AAG AAC ATA CAT AGG-3′
Sequence 2	5′-CCT ATG TAT GTT CTT TGT TGT-Cy3-3′	5′-CCT ATG TAT ATT CTT TGT TGT-Cy3-3′

## Data Availability

Data are contained within the article.

## Supplementary Materials

The following supporting information can be downloaded at https://www.mdpi.com/article/10.3390/s24186088/s1, Figure S1: Fluorescence optical signal from the DNA/dye hybridized silica microspheres.

## References

[1] Lander E.S., Linton L.M., Birren B., Nusbaum C., Zody M.C., Baldwin J., Devon K., Dewar K., Doyle M., FitzHugh W. (2001). Initial sequencing and analysis of the human genome. Nature.

[2] Higuchi R., von Beroldingen C.H., Sensabaugh G.F., Erlich H.A. (1988). DNA typing from single hairs. Nature.

[3] Jackson S.P., Bartek J. (2009). The DNA-damage response in human biology and disease. Nature.

[4] van ’t Veer L.J., Bernards R. (2008). Enabling personalized cancer medicine through analysis of gene-expression patterns. Nature.

[5] Lupski J.R. (1998). Genomic disorders: Structural features of the genome can lead to DNA rearrangements and human disease traits. Trends Genet..

[6] Pollack J.R., Perou C.M., Alizadeh A.A., Eisen M.B., Pergamenschikov A., Williams C.F., Jeffrey S.S., Botstein D., Brown P.O. (1999). Genome-wide analysis of DNA copy-number changes using cDNA microarrays. Nat. Genet..

[7] Robertson K.D. (2005). DNA methylation and human disease. Nat. Rev. Genet..

[8] Taylor R.W., Turnbull D.M. (2005). Mitochondrial DNA mutations in human disease. Nat. Rev. Genet..

[9] Shapiro B., Hofreiter M. (2014). A paleogenomic perspective on evolution and gene function: New insights from ancient DNA. Science.

[10] Shi H., Maier S., Nimmrich I., Yan P.S., Caldwell C.W., Olek A., Huang T.H. (2003). Oligonucleotide-based microarray for DNA methylation analysis: Principles and applications. J. Cell. Biochem..

[11] Cox W.G., Singer V.L. (2004). Fluorescent DNA hybridization probe preparation using amine modification and reactive dye coupling. BioTechniques.

[12] Reed G.H., Wittwer C.T. (2004). Sensitivity and specificity of single-nucleotide polymorphism scanning by high-resolution melting analysis. Clin. Chem..

[13] Canales R.D., Luo Y., Willey J.C., Austermiller B., Barbacioru C.C., Boysen C., Hunkapiller K., Jensen R.V., Knight C.R., Lee K.Y. (2006). Evaluation of DNA microarray results with quantitative gene expression platforms. Nat. Biotechnol..

[14] Montgomery J., Wittwer C.T., Palais R., Zhou L. (2007). Simultaneous mutation scanning and genotyping by high-resolution DNA melting analysis. Nat. Protoc..

[15] Zeglis B.M., Barton J.K. (2007). DNA base mismatch detection with bulky rhodium intercalators: Synthesis and applications. Nat. Protoc..

[16] Wu Z., Kou R., Ni K., Song R., Li Y., Li T., Zhang H. (2023). DNA Extraordinarily Stable Hairpin-Based Biosensors for Rapid Detection of DNA Ligases. Biosensors.

[17] Suter J.D., White I.M., Zhu H., Shi H., Caldwell C.W., Fan X. (2008). Label. Label-free quantitative DNA detection using the liquid core optical ring resonator. Biosens. Bioelectron..

[18] Sun Y., Shopova S.I., Wu C.S., Arnold S., Fan X. (2010). Bioinspired optofluidic FRET lasers via DNA scaffolds. Proc. Natl Acad. Sci. USA.

[19] Lee W., Fan X. (2012). Intracavity DNA melting analysis with optofluidic lasers. Anal. Chem..

[20] Sun Y., Fan X. (2012). Distinguishing DNA by analog-to-digital-like conversion by using optofluidic lasers. Angew. Chem. Int. Ed..

[21] Chen Q., Liu H., Lee W., Sun Y., Zhu D., Pei H., Fan C., Fan X. (2013). Self-assembled DNA tetrahedral optofluidic lasers with precise and tunable gain control. Lab Chip.

[22] Chen Q., Ritt M., Sivaramakrishnan S., Sun Y., Fan X. (2014). Optofluidic lasers with a single molecular layer of gain. Lab Chip.

[23] Lee W., Chen Q., Fan X., Yoon D.K. (2016). Digital DNA detection based on a compact optofluidic laser with ultra-low sample consumption. Lab Chip.

[24] Hou M., Liang X., Zhang T., Qiu C., Chen J., Liu S., Wang W., Fan X. (2018). DNA melting analysis with optofluidic lasers based on Fabry-Perot microcavity. ACS Sens..

[25] Shopova S.I., Zhou H., Fan X., Zhang P. (2007). Optofluidic ring resonator based dye laser. Appl. Phys. Lett..

[26] Lee W., Luo Y., Zhu Q., Fan X. (2011). Versatile optofluidic ring resonator lasers based on microdroplets. Opt. Express.

[27] Jun C.S., Lee W. (2022). High-throughput DNA analysis platform based on an optofluidic ring resonator laser. Appl. Sci..

[28] Li H., Guo Y., Sun Y., Reddy K., Fan X. (2010). Analysis of single nanoparticle detection by using 3-dimensionally confined optofluidic ring resonators. Opt. Express.

[29] Lee W., Sun Y., Li H., Reddy K., Sumetsky M., Fan X. (2011). A quasi-droplet optofluidic ring resonator laser using a micro-bubble. Appl. Phys. Lett..

