# Supplementary material for: Development of Lasing Silica Microsphere for High-Speed DNA Molecular Detection
Source: Sensors (Basel). 2024 Sep 20;24(18):6088. doi: 10.3390/s24186088 (PMC11435820; doi:10.3390/s24186088)
Supplement: Supplementary file 1 [file sensors-24-06088-s001.zip › sensors-3173163-supplementary.pdf]

sensors MDPI

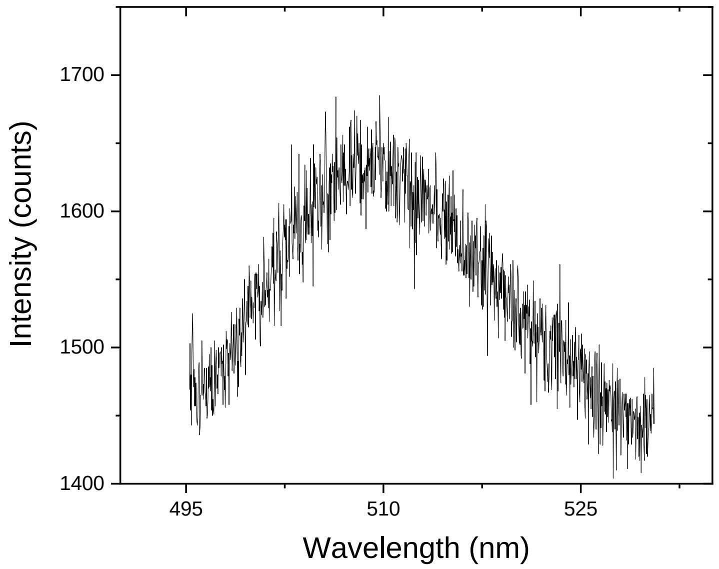

Figure S1. Fluorescence optical signal from the DNA/dye hybridized silica microspheres when optically pumped with the pump energy density below lasing threshold. Since the fluorescence optical signal is significantly lower than the laser signal, the spectrum is obtained with different settings such as shutter duration, numerical aperture, etc. Note that the fluorescence signal in the laser spectra is virtually zero.

.
